# Supplementary material for: Mendelian randomization uncovers a protective effect of interleukin-1 receptor antagonist on kidney function
Source: Commun Biol. 2023 Jul 14;6:722. doi: 10.1038/s42003-023-05091-8 (PMC10349143; doi:10.1038/s42003-023-05091-8)
Supplement: Supplementary file 2 — Supplementary information [file 42003_2023_5091_MOESM2_ESM.pdf]

Supplementary information

Mendelian randomization uncovers a protective effect of interleukin-1 receptor antagonist on kidney function

Authors

Jeong Min Cho, Jung Hun Koh, Seong Geun Kim, Soojin Lee, Yaerim Kim, Semin Cho, Kwangsoo Kim, Yong Chul Kim, Seung Seok Han, Hajeong Lee, Jung Pyo Lee, Kwon Wook Joo, Chun Soo Lim, Yon Su Kim, Dong Ki Kim, and Sehoon Park

Supplementary information table of contents

Supplementary Note 1. Cis-instrumental selection criteria.

Supplementary Table 1. Summary-level MR results of genetically predicted serum IL levels using cis-pQTL and cis-eQTL instruments with CKDGen data.

Supplementary Table 2. Sensitivity analysis by excluding cis-eQTL instruments.

Supplementary Table 3. Sensitivity analysis by excluding palindromic SNPs.

Supplementary Table 4. Minimum effect size for 80% power in each IL

Supplementary Table 5. Strengths of cis-pQTL and cis-eQTL instruments per interleukin.

Supplementary Table 6. Genetic instruments of interleukins included in the Mendelian randomization analysis.

Supplementary References

Supplemental Note 1. Cis-instrumental selection criteria.

Two cis-instrumental selection criteria were used to identify variants that proxy the effect of circulating cytokine levels in MR analyses: (1) a cis-pQTL criteria, involving genetic variants located within 500 kb of the gene locus corresponding to a cytokine, that associated with the circulating cytokine concentrations at P value $<1 \times 10^{-4}$, and (2) a cis-eQTL criteria, involving variants located within 500 kb of the gene locus corresponding to a cytokine, that associated with both its gene expression at P $<1 \times 10^{-4}$ and its circulating cytokine concentrations at P <0.05. The strength of the cis-genetic instruments was identified by measuring the median explained variance (r^2) for each genetic variant based on the circulating cytokine concentrations.

Supplemental Table 1. Summary-level MR results of genetically predicted serum IL levels using cis-pQTL and cis-eQTL instruments with CKDGen data.

Interleukin	[a]Outcome	MR-Egger intercept P	MR methods	eGFR change beta (%)	Standard error (%)	P value
IL-1α	1) Creatinine-based log-eGFR values (CKDGen + UKB)	5E-01	MR-IVW	-0.32	1.21	0.789
			MR-Egger	-0.58	9.63	0.45
			Weighed median	-1.01	1.82	0.574
	2) Creatinine-based log-eGFR values (CKDGen)	4E-01	MR-IVW	-0.39	1.18	0.736
			MR-Egger	-0.09	12.52	0.442
			Weighed median	-1.18	7.86	0.876
	3) Cystatin-C-based log-eGFR values (CKDGen + UKB)	5E-01	MR-IVW	-0.05	1.40	0.972
			MR-Egger	-0.19	10.85	0.457
			Weighed median	-0.91	4.06	0.819
	4) Degree of annual eGFR decline (CKDGen + UKB)	5E-01	MR-IVW	7.24	11.37	0.516
			MR-Egger	4.00	120.68	0.474
			Weighed median	9.29	24.39	0.684
IL-2ra	1) Creatinine-based log-eGFR values (CKDGen + UKB)	0.44	MR-IVW	0.13	0.06	0.028
			MR-Egger	-0.04	0.10	0.314
			Weighed median	0.10	0.07	0.167
	2) Creatinine-based log-eGFR values (CKDGen)	0.50	MR-IVW	0.08	0.05	0.133
			MR-Egger	0.07	0.13	0.293
			Weighed median	0.10	0.08	0.261
	3) Cystatin-C-based log-eGFR values (CKDGen + UKB)	0.03	MR-IVW	0.18	0.10	0.052
			MR-Egger	0.03	0.19	0.432
			Weighed median	0.05	0.11	0.669
	4) Degree of annual eGFR decline	0.78	MR-IVW	-0.02	0.63	0.970
			MR-Egger	0.15	1.63	0.466

	(CKDGen + UKB)		Weighed median	0.04	1.04	0.967
IL-16	1) Creatinine-based log-eGFR values (CKDGen + UKB)	0.88	MR-IVW	0.05	0.04	0.150
			MR-Egger	0.08	0.07	0.153
			Weighed median	0.05	0.07	0.423
	2) Creatinine-based log-eGFR values (CKDGen)	0.81	MR-IVW	0.05	0.06	0.402
			MR-Egger	0.01	0.14	0.484
			Weighed median	0.09	0.11	0.411
	3) Cystatin-C-based log-eGFR values (CKDGen + UKB)	0.57	MR-IVW	0.07	0.10	0.509
			MR-Egger	0.09	0.18	0.321
			Weighed median	0.07	0.15	0.638
	4) Degree of annual eGFR decline (CKDGen + UKB)	0.07	MR-IVW	-0.55	1.21	0.648
			MR-Egger	1.48	1.71	0.207
			Weighed median	0.32	1.35	0.814
IL-18	1) Creatinine-based log-eGFR values (CKDGen + UKB)	0.70	MR-IVW	0.03	0.05	0.540
			MR-Egger	-0.03	0.17	0.413
			Weighed median	-0.01	0.10	0.897
	2) Creatinine-based log-eGFR values (CKDGen)	0.36	MR-IVW	-0.04	0.07	0.590
			MR-Egger	-0.14	0.23	0.262
			Weighed median	-0.09	0.13	0.493
	3) Cystatin-C-based log-eGFR values (CKDGen + UKB)	0.43	MR-IVW	0.12	0.06	0.033
			MR-Egger	-0.03	0.28	0.447
			Weighed median	0.06	0.16	0.699
	4) Degree of annual eGFR decline (CKDGen + UKB)	0.84	MR-IVW	-1.27	0.51	0.011
			MR-Egger	-1.03	2.84	0.346
			Weighed median	-1.39	1.58	0.372
IL-8	1) Creatinine-based log-eGFR values (CKDGen + UKB)	NA	MR-IVW	-0.14	0.05	0.009
	2) Creatinine-based log-eGFR values	NA	MR-IVW	-0.21	0.58	0.713
	3) Cystatin-C-based	NA	MR-IVW	0.17	0.04	1E-05

	log-eGFR values					
	4) Degree of annual eGFR decline (CKDGen + UKB)	NA	MR-IVW	-3.58	9.56	0.690
[b]*IL-6	1) Creatinine-based log-eGFR values (CKDGen + UKB)	NA	Ratio of coefficient	0.29	0.89	0.739
	2) Creatinine-based log-eGFR values	NA	Ratio of coefficient	0.52	1.01	0.606
	3) Cystatin-C-based log-eGFR values	NA	Ratio of coefficient	-5.99	1.48	3E-05
	4) Degree of annual eGFR decline (CKDGen + UKB)	NA	Ratio of coefficient	28.40	13.82	0.053
*IL-7	1) Creatinine-based log-eGFR values (CKDGen + UKB)	NA	Ratio of coefficient	0.10	0.31	0.739
	2) Creatinine-based log-eGFR values	NA	Ratio of coefficient	-0.30	0.45	0.510
	3) Cystatin-C-based log-eGFR values	NA	Ratio of coefficient	-0.41	0.51	0.424
	4) Degree of annual eGFR decline (CKDGen + UKB)	NA	Ratio of coefficient	-2.22	5.13	0.653
*IL-12p70	1) Creatinine-based log-eGFR values (CKDGen + UKB)	NA	Ratio of coefficient	5.04	6.31	0.422
	2) Creatinine-based log-eGFR values	NA	Ratio of coefficient	-0.43	0.52	0.399
	3) Cystatin-C-based log-eGFR values	NA	Ratio of coefficient	-0.26	0.61	0.688
	4) Degree of annual	NA	Ratio of coefficient	5.04	6.31	0.422

	eGFR decline (CKDGen + UKB)					

MR = Mendelian randomization, eGFR = estimated glomerular filtration rate, MR-IVW = multiplicative random-effects inverse variance-weighted
Both cis-pQTL and cis-eQTL instruments were included as genetic instruments of genetically predicted serum IL level.
[a]Four summary statistics for kidney function traits were utilized for MR analyses: (from the first row) 1) creatinine-based log-eGFR values of the CKDGen and the UKB data,[1] 2) creatinine-based log-eGFR values from the phase 4 CKDGen study,[2] 3) cystatin C-based log-eGFR values from CKDGen and UKB,[1] and 4) eGFR decline including CKDGen and UKB.[3]
[b]A cis-pQTL instrument of IL-6 (rs57349960) was not included in the analysis because the summary statistics were unavailable in the outcome GWAS databases.
*When only a single genetic instrument was provided, the ratio of coefficient (Wald ratio) method was applied to measure MR estimates. MR-Egger intercept P value was unable to be calculated and presented as NA.
All effect sizes were aligned and scaled towards genetically predicted standard deviation increase in serum IL concentration for % change in eGFR.

Supplemental Table 2. Sensitivity analysis by excluding cis-eQTL instruments.

Interleukin	[a]Outcome	MR-Egger intercept P	MR methods	eGFR change beta (%)	Standard error (%)	P value
IL-1ra	1) Creatinine-based log-eGFR values (CKDGen + UKB)	0.84	MR-IVW	0.28	0.11	0.009
			MR-Egger	0.36	0.13	0.003
			Weighed median	0.32	0.11	0.005
	2) Creatinine-based log-eGFR values (CKDGen)	0.53	MR-IVW	0.32	0.09	3E-04
			MR-Egger	0.25	0.14	0.034
			Weighed median	0.36	0.12	0.003
	3) Cystatin-C-based log-eGFR values (CKDGen + UKB)	0.16	MR-IVW	0.35	0.17	0.039
			MR-Egger	0.75	0.21	0.001
			Weighed median	0.52	0.17	0.003
	4) Degree of annual eGFR decline (CKDGen + UKB)	0.29	MR-IVW	-2.18	1.09	0.043
			MR-Egger	-2.40	1.66	0.068
			Weighed median	-2.85	1.50	0.052
IL-2ra	1) Creatinine-based log-eGFR values (CKDGen + UKB)	0.60	MR-IVW	0.12	0.06	0.039
			MR-Egger	-0.03	0.10	0.398
			Weighed median	0.09	0.09	0.292
	2) Creatinine-based log-eGFR values (CKDGen)	0.38	MR-IVW	0.07	0.05	0.172
			MR-Egger	0.11	0.13	0.201
			Weighed median	0.09	0.09	0.292
	3) Cystatin-C-based log-eGFR values (CKDGen + UKB)	0.05	MR-IVW	0.17	0.10	0.072
			MR-Egger	0.05	0.19	0.4
			Weighed median	0.05	0.10	0.652
	4) Degree of annual eGFR decline (CKDGen + UKB)	0.74	MR-IVW	-0.01	0.66	0.989
			MR-Egger	0.11	1.66	0.471
			Weighed median	0.08	1.03	0.937
IL-16	1) Creatinine-based log-eGFR values (CKDGen + UKB)	0.78	MR-IVW	0.03	0.05	0.631
			MR-Egger	0.05	0.10	0.293
			Weighed median	0.04	0.09	0.670

	2) Creatinine-based log-eGFR values (CKDGen)	0.66	MR-IVW	0.04	0.09	0.641
			MR-Egger	-0.05	0.21	0.43
			Weighed median	0.10	0.14	0.502
	3) Cystatin-C-based log-eGFR values (CKDGen + UKB)	0.34	MR-IVW	0.02	0.16	0.922
			MR-Egger	0.13	0.26	0.311
			Weighed median	0.03	0.19	0.893
	4) Degree of annual eGFR decline (CKDGen + UKB)	0.33	MR-IVW	-0.49	1.48	0.737
			MR-Egger	0.67	2.32	0.384
			Weighed median	0.33	1.70	0.845
IL-18	1) Creatinine-based log-eGFR values (CKDGen + UKB)	NA	MR-IVW	0.03	0.05	0.504
			MR-Egger	-0.09	0.18	0.318
			Weighed median	-0.01	0.09	0.904
	2) Creatinine-based log-eGFR values (CKDGen)	0.44	MR-IVW	-0.05	0.07	0.527
			MR-Egger	-0.10	0.25	0.343
			Weighed median	-0.09	0.12	0.467
	3) Cystatin-C-based log-eGFR values (CKDGen + UKB)	NA	MR-IVW	0.11	0.04	0.012
			MR-Egger	0.001	0.29	0.499
			Weighed median	0.06	0.16	0.703
	4) Degree of annual eGFR decline (CKDGen + UKB)	NA	MR-IVW	-1.22	0.55	0.024
			MR-Egger	-1.81	3.16	0.283
			Weighed median	-1.37	1.68	0.407
*IL-7	1) Creatinine-based log-eGFR values (CKDGen + UKB)	NA	Ratio of coefficient	0.10	0.31	0.739
	2) Creatinine-based log-eGFR values	NA	Ratio of coefficient	-0.30	0.45	0.510
	3) Cystatin-C-based log-eGFR values	NA	Ratio of coefficient	-0.41	0.51	0.424
	4) Degree of annual eGFR decline (CKDGen + UKB)	NA	Ratio of coefficient	-2.22	5.13	0.653

*IL-8	1) Creatinine-based log-eGFR values (CKDGen + UKB)	NA	Ratio of coefficient	0.21	5.29	0.967
	2) Creatinine-based log-eGFR values	NA	Ratio of coefficient	0.04	0.44	0.918
	3) Cystatin-C-based log-eGFR values	NA	Ratio of coefficient	0.16	0.53	0.764
	4) Degree of annual eGFR decline (CKDGen + UKB)	NA	Ratio of coefficient	0.21	5.29	0.967
*IL-12p70	1) Creatinine-based log-eGFR values (CKDGen + UKB)	NA	Ratio of coefficient	5.04	6.31	0.422
	2) Creatinine-based log-eGFR values	NA	Ratio of coefficient	-0.43	0.52	0.399
	3) Cystatin-C-based log-eGFR values	NA	Ratio of coefficient	-0.26	0.61	0.668
	4) Degree of annual eGFR decline (CKDGen + UKB)	NA	Ratio of coefficient	5.04	6.31	0.422

MR = Mendelian randomization, eGFR = estimated glomerular filtration rate, MR-IVW = multiplicative random-effects inverse variance-weighted
[a]Four summary statistics for kidney function traits were utilized for MR analyses: (from the first row) 1) creatinine-based log-eGFR values of the CKDGen and the UKB data,[1] 2) creatinine-based log-eGFR values from the phase 4 CKDGen study,[2] 3) cystatin C-based log-eGFR values from CKDGen and UKB,[1] and 4) eGFR decline including CKDGen and UKB.[3]
*When only a single genetic instrument was provided, the ratio of coefficient (Wald ratio) method was applied to measure MR estimates. MR-Egger intercept P value was unable to be calculated and presented as NA.
IL-1α was not included in the analysis because only cis-eQTL instruments were provided for IL-1α. IL-6 was also not included in the analysis because the summary statistics for cis-pQTL instrument (rs57349960) were unavailable in the outcome GWAS databases.
Genetic variant of IL-6 was not included in the analysis because the summary statistics were unavailable in the outcome GWAS databases.
All effect sizes were aligned and scaled towards genetically predicted standard deviation increase in serum IL concentration for % change in eGFR.

Supplemental Table 3. Sensitivity analysis by excluding palindromic SNPs.

Interleukin	[a]Outcome	MR-Egger intercept P	MR methods	eGFR change beta (%)	Standard error (%)	P value
IL-1α	1) Creatinine-based log-eGFR values (CKDGen + UKB)	0.46	MR-IVW	-0.25	1.28	0.841
			MR-Egger	-0.11	10.43	0.451
			Weighed median	-1.04	3.31	0.748
	2) Creatinine-based log-eGFR values (CKDGen)	0.42	MR-IVW	-0.39	1.18	0.736
			MR-Egger	-0.41	10.82	0.421
			Weighed median	-1.18	4.44	0.785
	3) Cystatin-C-based log-eGFR values (CKDGen + UKB)	0.47	MR-IVW	-0.05	1.40	0.972
			MR-Egger	-0.81	27.10	0.468
			Weighed median	-0.91	3.27	0.777
	4) Degree of annual eGFR decline (CKDGen + UKB)	0.46	MR-IVW	7.24	11.37	0.516
			MR-Egger	2.29	136.01	0.501
			Weighed median	9.29	272.03	0.946
IL-1ra	1) Creatinine-based log-eGFR values (CKDGen + UKB)	0.84	MR-IVW	0.28	0.11	0.009
			MR-Egger	0.36	0.13	0.003
			Weighed median	0.32	0.11	0.005
	2) Creatinine-based log-eGFR values (CKDGen)	0.53	MR-IVW	0.32	0.09	3E-04
			MR-Egger	0.25	0.14	0.034
			Weighed median	0.36	0.12	0.003
	3) Cystatin-C-based log-eGFR values (CKDGen + UKB)	0.16	MR-IVW	0.35	0.17	0.039
			MR-Egger	0.75	0.21	0.001
			Weighed median	0.52	0.17	0.003
	4) Degree of annual eGFR decline (CKDGen + UKB)	0.29	MR-IVW	-2.18	1.09	0.043
			MR-Egger	-2.40	1.66	0.068
			Weighed median	-2.85	1.50	0.052
IL-2ra	1) Creatinine-based log-eGFR values (CKDGen + UKB)	0.44	MR-IVW	0.13	0.06	0.028
			MR-Egger	-0.04	0.10	0.324
			Weighed median	0.10	0.07	0.150

	2) Creatinine-based log-eGFR values (CKDGen)	0.50	MR-IVW	0.08	0.05	0.133
			MR-Egger	0.07	0.13	0.286
			Weighed median	0.10	0.09	0.268
	3) Cystatin-C-based log-eGFR values (CKDGen + UKB)	0.03	MR-IVW	0.18	0.10	0.052
			MR-Egger	0.02	0.18	0.439
			Weighed median	0.05	0.10	0.662
	4) Degree of annual eGFR decline (CKDGen + UKB)	0.78	MR-IVW	-0.02	0.63	0.970
			MR-Egger	0.07	1.63	0.475
			Weighed median	0.04	1.09	0.968
IL-16	1) Creatinine-based log-eGFR values (CKDGen + UKB)	0.88	MR-IVW	0.05	0.04	0.150
			MR-Egger	0.07	0.07	0.158
			Weighed median	0.05	0.07	0.409
	2) Creatinine-based log-eGFR values (CKDGen)	0.81	MR-IVW	0.05	0.06	0.402
			MR-Egger	0.02	0.14	0.454
			Weighed median	0.09	0.11	0.423
	3) Cystatin-C-based log-eGFR values (CKDGen + UKB)	0.57	MR-IVW	0.07	0.10	0.509
			MR-Egger	0.08	0.17	0.321
			Weighed median	0.07	0.14	0.616
	4) Degree of annual eGFR decline (CKDGen + UKB)	0.07	MR-IVW	-0.55	1.21	0.648
			MR-Egger	1.50	1.71	0.182
			Weighed median	0.32	1.36	0.815
IL-18	1) Creatinine-based log-eGFR values (CKDGen + UKB)	0.70	MR-IVW	0.03	0.05	0.540
			MR-Egger	-0.04	0.17	0.403
			Weighed median	-0.01	0.10	0.900
	2) Creatinine-based log-eGFR values (CKDGen)	0.36	MR-IVW	-0.04	0.07	0.590
			MR-Egger	-0.13	0.22	0.280
			Weighed median	-0.09	0.13	0.509
	3) Cystatin-C-based log-eGFR values (CKDGen + UKB)	0.43	MR-IVW	0.12	0.06	0.033
			MR-Egger	-0.06	0.28	0.417
			Weighed median	0.06	0.15	0.685
	4) Degree of annual	0.84	MR-IVW	-1.27	0.51	0.011

	eGFR decline (CKDGen + UKB)		MR-Egger	-1.03	2.83	0.350
			Weighed median	-1.39	1.58	0.374
IL-8	1) Creatinine-based log-eGFR values (CKDGen + UKB)	NA	MR-IVW	-0.14	0.05	0.009
	2) Creatinine-based log-eGFR values	NA	MR-IVW	-0.21	0.58	0.713
	3) Cystatin-C-based log-eGFR values	NA	MR-IVW	0.17	0.04	1E-05
	4) Degree of annual eGFR decline (CKDGen + UKB)	NA	MR-IVW	-3.58	9.56	0.690
*IL-6	1) Creatinine-based log-eGFR values (CKDGen + UKB)	NA	Ratio of coefficient	0.29	0.89	0.739
	2) Creatinine-based log-eGFR values	NA	Ratio of coefficient	0.52	1.01	0.606
	3) Cystatin-C-based log-eGFR values	NA	Ratio of coefficient	**-	-	-
	4) Degree of annual eGFR decline (CKDGen + UKB)	NA	Ratio of coefficient	-	-	-
*IL-7	1) Creatinine-based log-eGFR values (CKDGen + UKB)	NA	Ratio of coefficient	1E-03	3E-03	0.739
	2) Creatinine-based log-eGFR values	NA	Ratio of coefficient	-3E-03	5E-03	0.510
	3) Cystatin-C-based log-eGFR values	NA	Ratio of coefficient	-4E-03	5E-03	0.424
	4) Degree of annual eGFR decline (CKDGen + UKB)	NA	Ratio of coefficient	-0.02	0.05	0.653

*IL-12p70	1) Creatinine-based log-eGFR values (CKDGen + UKB)	NA	Ratio of coefficient	-5E-03	3E-03	0.134
	2) Creatinine-based log-eGFR values	NA	Ratio of coefficient	-4E-03	0.01	0.399
	3) Cystatin-C-based log-eGFR values	NA	Ratio of coefficient	-3E-03	6E-03	0.668
	4) Degree of annual eGFR decline (CKDGen + UKB)	NA	Ratio of coefficient	0.05	0.06	0.422

SNP = single nucleotide polymorphism, MR = Mendelian randomization, eGFR = estimated glomerular filtration rate, MR-IVW = multiplicative random-effects inverse variance-weighted

[a]Four summary statistics for kidney function traits were utilized for MR analyses: (from the first row) 1) creatinine-based log-eGFR values of the CKDGen and the UKB data,[1] 2) creatinine-based log-eGFR values from the phase 4 CKDGen study,[2] 3) cystatin C-based log-eGFR values from CKDGen and UKB,[1] and 4) eGFR decline including CKDGen and UKB.[3]

*When only a single genetic instrument was provided, the ratio of coefficient method (Wald ratio) was applied to measure MR estimates. MR-Egger intercept P value was unable to be calculated and presented as NA.

**In IL-6, MR analysis with cystatin-C based log-eGFR values and eGFR decline summary statistics were not available after excluding a palindromic SNP.

All effect sizes were aligned and scaled towards genetically predicted standard deviation increase in serum IL concentration for % change in eGFR.

Supplemental Table 4. Minimum effect size for 80% power in each IL

Interleukin	pQTL		eQTL		pQTL and eQTL	
	r^2	Minimum effect size	r^2	Minimum effect size	r^2	Minimum effect size
IL-1α	–	–	0.003	0.041	0.003	0.041
IL-1ra	0.075	0.008	0.017	0.017	0.092	0.007
IL-2ra	0.260	0.02	0.130	0.028	0.390	0.003
IL-6	0.002	0.051	0.001	0.071	0.003	0.041
IL-7	0.005	0.032	–	–	0.005	0.032
IL-8	0.004	0.036	0.005	0.032	0.009	0.024
IL-12p70	0.002	0.051	–	–	0.002	0.051
IL-16	0.037	0.012	0.031	0.013	0.068	0.008
IL-18	0.051	0.01	0.024	0.015	0.075	0.008

IL = interleukin, QTL = quantitative trait loci
Effect size was calculated in https://shiny.cnsgenomics.com/mRnd/. CKDGen + UKB meta-analysis of creatinine-based log-eGFR values was used as outcome dataset and type-I error rate was set at 0.25.

Supplemental Table 5. Strengths of cis-pQTL and cis-eQTL instruments per interleukin.

Interleukin	Gene	cis-pQTL (*n* instruments)	pQTL (r^2)	cis-eQTL (*n* instruments)	eQTL (r^2)
IL-1α	*IL1A*	–	–	3	0.003
IL-1ra	*IL1RN*	18	0.075	2	0.017
IL-2ra	*IL2RA*	14	0.260	4	0.130
IL-6	*IL6*	1	0.002	1	0.001
IL-7	*IL7*	1	0.005	–	–
IL-8	*CXCL8*	1	0.004	2	0.005
IL-12p70	*IL12A*	1	0.002	–	–
IL-12p70	*IL12B*	1	0.002	–	–
IL-16	*IL16*	18	0.037	6	0.031
IL-18	*IL18*	5	0.051	2	0.024

IL = interleukin, QTL = quantitative trait loci

Supplemental Table 6. Genetic instruments of interleukins included in the Mendelian randomization analysis.

Interleukin	RSID	Chr	Chromosome start	EA	NEA	Beta coefficient	Standard error	P value	R^2	F-statistics	Instrument
IL-1α	rs895497	2	113763575	G	A	0.048	0.0243	4.9E-02	0.001	3.568	eQTL
IL-1α	rs1533463	2	113538782	A	G	0.046	0.0205	2.6E-02	0.001	5.085	eQTL
IL-1α	rs1143643	2	113588302	T	C	-0.050	0.0224	2.4E-02	0.001	5.350	eQTL
IL-1ra	rs9308676	2	113428167	A	G	0.043	0.0109	7.4E-05	0.001	17.500	pQTL
IL-1ra	rs7594852	2	113521754	T	C	0.045	0.0103	1.3E-05	0.001	19.191	pQTL
IL-1ra	rs45592842	2	113943063	T	G	0.095	0.0230	3.8E-05	0.001	20.807	pQTL
IL-1ra	rs3783548	2	113533333	A	G	0.086	0.0207	3.4E-05	0.001	20.963	pQTL
IL-1ra	rs62157451	2	113608418	C	G	-0.096	0.0226	2.3E-05	0.001	20.979	pQTL
IL-1ra	rs6723639	2	113872238	T	C	-0.066	0.0133	7.8E-07	0.001	26.848	pQTL
IL-1ra	rs72831808	2	113979355	C	G	0.124	0.0252	9.7E-07	0.002	27.117	pQTL
IL-1ra	rs3977064	2	114191728	A	C	-0.104	0.0243	2.1E-05	0.002	29.292	pQTL
IL-1ra	rs1191686	2	114094703	T	C	-0.072	0.0131	4.3E-08	0.002	30.009	pQTL
IL-1ra	rs1867868	2	114162983	T	C	0.063	0.0106	2.2E-09	0.002	36.564	pQTL
IL-1ra	rs4591347	2	113658705	T	C	-0.101	0.0149	1.7E-11	0.002	45.084	pQTL
IL-1ra	rs1491585	2	114003621	A	G	-0.075	0.0107	2.0E-12	0.003	53.891	pQTL
IL-1ra	rs7584409	2	113742734	A	G	-0.086	0.0116	9.5E-14	0.003	55.400	pQTL
IL-1ra	rs114189538	2	113821082	A	G	-0.167	0.0222	6.2E-14	0.003	65.429	pQTL
IL-1ra	rs74667587	2	113895079	A	G	-0.149	0.0141	2.7E-26	0.007	126.716	pQTL
IL-1ra	rs72829856	2	113913287	A	G	0.213	0.0196	3.0E-27	0.008	142.537	pQTL
IL-1ra	rs6743376	2	113832333	A	C	-0.182	0.0107	3.5E-65	0.015	293.317	pQTL
IL-1ra	rs6734238	2	113841030	A	G	0.204	0.0104	2.5E-85	0.020	384.119	pQTL
IL-1ra	rs315930	2	113869554	A	C	-0.035	0.0111	1.7E-03	0.001	10.156	eQTL

IL-1ra	rs11677088	2	113835603	A	G	0.183	0.0103	6.0E-71	0.016	319.307	eQTL
IL-2ra	rs3136613	10	6020749	C	T	0.096	0.0240	6.4E-05	0.004	15.412	pQTL
IL-2ra	rs7084255	10	6184167	T	A	0.134	0.0304	1.0E-05	0.005	19.277	pQTL
IL-2ra	rs4747891	10	6188119	G	C	0.180	0.0375	1.6E-06	0.006	20.753	pQTL
IL-2ra	rs7077067	10	6132692	C	T	0.111	0.0240	3.7E-06	0.006	21.983	pQTL
IL-2ra	rs676063	10	5986900	G	T	0.146	0.0354	3.7E-05	0.006	22.481	pQTL
IL-2ra	rs7898286	10	6009462	G	T	-0.119	0.0252	2.5E-06	0.007	24.358	pQTL
IL-2ra	rs2182409	10	6209218	C	T	-0.120	0.0249	1.5E-06	0.007	25.369	pQTL
IL-2ra	rs3136614	10	6005674	G	A	0.165	0.0363	5.3E-06	0.008	30.084	pQTL
IL-2ra	rs9423654	10	5605334	G	C	0.135	0.0283	1.7E-06	0.008	30.918	pQTL
IL-2ra	rs17403348	10	5827901	C	T	0.334	0.0805	3.4E-05	0.013	47.167	pQTL
IL-2ra	rs140148883	10	5795575	G	A	-0.402	0.0937	1.8E-05	0.016	57.973	pQTL
IL-2ra	rs12722588	10	6060433	C	T	0.334	0.0334	1.6E-23	0.026	96.209	pQTL
IL-2ra	rs34507893	10	6124598	G	A	-0.735	0.1155	2.0E-10	0.057	220.921	pQTL
IL-2ra	rs12722497	10	6095928	C	A	-0.629	0.0482	6.9E-39	0.093	376.999	pQTL
IL-2ra	rs8177751	10	6029759	T	A	0.108	0.0384	4.8E-03	0.003	11.697	eQTL
IL-2ra	rs3136614	10	6005674	G	A	0.165	0.0363	5.3E-06	0.008	30.108	eQTL
IL-2ra	rs12722588	10	6060433	C	T	0.334	0.0334	1.6E-23	0.025	96.009	eQTL
IL-2ra	rs12722497	10	6095928	C	A	-0.629	0.0482	6.9E-39	0.093	376.065	eQTL
IL-6	rs57349960	7	22519455	CG	C	0.061	0.0153	6.3E-05	0.002	16.085	pQTL
IL-6	rs7808457	7	22798265	A	T	-0.034	0.0118	4.0E-03	0.001	8.106	eQTL
IL-7	rs6473130	8	79862570	C	A	-0.098	0.0251	9.7E-05	0.005	16.088	pQTL
IL-8	rs7655660	4	74589597	A	G	-0.188	0.0237	2.7E-15	0.004	71.867	pQTL
IL-8	rs28833215	4	74646006	A	G	-0.036	0.0118	2.5E-03	0.001	10.313	eQTL
IL-8	rs7655660	4	74589597	A	G	-0.188	0.0237	2.7E-15	0.004	71.867	eQTL

IL12p70	rs34740816	3	159965219	G	A	-0.116	0.0296	8.2E-05	0.002	NA	pQTL
IL-16	rs7170924	15	81583139	T	G	0.024	0.0051	4.1E-06	0.000	4.525	pQTL
IL-16	rs12913587	15	81836114	A	G	-0.022	0.0051	1.7E-05	0.000	5.044	pQTL
IL-16	rs1848707	15	81421792	A	G	-0.024	0.0047	3.1E-07	0.000	5.525	pQTL
IL-16	rs1482937	15	81811499	C	G	-0.028	0.0060	2.8E-06	0.000	5.850	pQTL
IL-16	rs75194383	15	81664543	T	G	0.053	0.0126	2.7E-05	0.000	6.531	pQTL
IL-16	rs7179124	15	81960730	C	G	-0.027	0.0050	5.8E-08	0.000	6.866	pQTL
IL-16	rs6495562	15	81612117	T	C	-0.061	0.0108	1.4E-08	0.000	8.395	pQTL
IL-16	rs116947254	15	81786092	A	G	-0.057	0.0123	3.3E-06	0.000	8.488	pQTL
IL-16	rs35000575	15	81439638	A	C	-0.045	0.0076	3.8E-09	0.000	9.592	pQTL
IL-16	rs4611404	15	81613766	T	C	-0.068	0.0118	8.6E-09	0.000	9.658	pQTL
IL-16	rs58484457	15	81769674	T	C	0.035	0.0048	2.5E-13	0.000	10.543	pQTL
IL-16	rs35254208	15	81481945	A	T	-0.041	0.0077	1.4E-07	0.001	10.649	pQTL
IL-16	rs4128767	15	81543407	A	G	-0.051	0.0047	2.1E-27	0.001	23.911	pQTL
IL-16	rs4778893	15	81611253	A	G	-0.063	0.0067	3.9E-21	0.001	25.768	pQTL
IL-16	rs55889255	15	81516565	A	G	0.103	0.0091	5.4E-30	0.002	35.414	pQTL
IL-16	rs75824993	15	81494541	A	C	-0.108	0.0092	9.0E-32	0.002	36.929	pQTL
IL-16	rs3848180	15	81596590	T	G	0.099	0.0041	1.1E-129	0.005	103.712	pQTL
IL-16	rs4778890	15	81589648	C	G	0.448	0.0089	0.0E+00	0.022	473.635	pQTL
IL-16	rs7164258	15	81661346	A	G	-0.022	0.0056	9.4E-05	0.000	3.899	eQTL
IL-16	rs3922930	15	81630421	A	G	-0.029	0.0057	4.4E-07	0.000	6.414	eQTL
IL-16	rs11635456	15	81524488	A	G	0.064	0.0086	1.5E-13	0.001	15.841	eQTL
IL-16	rs8023541	15	81577808	T	C	-0.051	0.0042	1.4E-33	0.001	27.207	eQTL
IL-16	rs11325	15	81601340	T	G	-0.080	0.0070	4.3E-30	0.001	31.184	eQTL
IL-16	rs4778639	15	81600451	T	G	0.459	0.0083	0.0E+00	0.027	597.649	eQTL

IL-18	rs76785871	11	112387246	A	G	-0.149	0.0369	5.5E-05	0.003	23.097	pQTL
IL-18	rs10789893	11	112391048	T	C	0.113	0.0247	4.6E-06	0.005	38.034	pQTL
IL-18	rs55772679	11	112235908	T	C	-0.134	0.0239	2.2E-08	0.009	62.039	pQTL
IL-18	rs1534115	11	112210999	A	G	-0.149	0.0251	3.3E-09	0.010	69.511	pQTL
IL-18	rs71478720	11	112009605	T	C	-0.260	0.0273	1.7E-21	0.023	163.445	pQTL
IL-18	rs2853127	11	112140332	T	C	-0.055	0.0255	3.2E-02	0.001	8.472	eQTL
IL-18	rs71478720	11	112009605	T	C	-0.260	0.0273	1.7E-21	0.023	163.611	eQTL

EA = effect allele, NEA = non-effect allele

Supplementary References

1. Stanzick KJ, Li Y, Schlosser P, et al. Discovery and prioritization of variants and genes for kidney function in >1.2 million individuals. Nat Commun 2021;12(1):4350. (In eng). DOI: 10.1038/s41467-021-24491-0.
2. Wuttke M, Li Y, Li M, et al. A catalog of genetic loci associated with kidney function from analyses of a million individuals. Nat Genet 2019;51(6):957-972. (In eng). DOI: 10.1038/s41588-019-0407-x.
3. Gorski M, Rasheed H, Teumer A, et al. Genetic loci and prioritization of genes for kidney function decline derived from a meta-analysis of 62 longitudinal genome-wide association studies. Kidney Int 2022;102(3):624-639. (In eng). DOI: 10.1016/j.kint.2022.05.021.
