# Supplementary material for: Mendelian randomization uncovers a protective effect of interleukin-1 receptor antagonist on kidney function
Source: Commun Biol. 2023 Jul 14;6:722. doi: 10.1038/s42003-023-05091-8 (PMC10349143; doi:10.1038/s42003-023-05091-8)
Supplement: Supplementary file 3 — Reporting Summary [file 42003_2023_5091_MOESM3_ESM.pdf]

nature portfolio

Corresponding author(s): Sehoon Park

Last updated by author(s): 2023.06.21

Reporting Summary

Nature Portfolio wishes to improve the reproducibility of the work that we publish. This form provides structure for consistency and transparency in reporting. For further information on Nature Portfolio policies, see our Editorial Policies and the Editorial Policy Checklist.

Statistics

For all statistical analyses, confirm that the following items are present in the figure legend, table legend, main text, or Methods section.

n/a	Confirmed	
☐	☒	The exact sample size (*n*) for each experimental group/condition, given as a discrete number and unit of measurement
☐	☒	A statement on whether measurements were taken from distinct samples or whether the same sample was measured repeatedly
☐	☒	The statistical test(s) used AND whether they are one- or two-sided<br>*Only common tests should be described solely by name; describe more complex techniques in the Methods section.*
☐	☒	A description of all covariates tested
☐	☒	A description of any assumptions or corrections, such as tests of normality and adjustment for multiple comparisons
☐	☒	A full description of the statistical parameters including central tendency (e.g. means) or other basic estimates (e.g. regression coefficient) AND variation (e.g. standard deviation) or associated estimates of uncertainty (e.g. confidence intervals)
☐	☒	For null hypothesis testing, the test statistic (e.g. *F*, *t*, *r*) with confidence intervals, effect sizes, degrees of freedom and *P* value noted<br>*Give P values as exact values whenever suitable.*
☒	☐	For Bayesian analysis, information on the choice of priors and Markov chain Monte Carlo settings
☐	☒	For hierarchical and complex designs, identification of the appropriate level for tests and full reporting of outcomes
☐	☒	Estimates of effect sizes (e.g. Cohen's *d*, Pearson's *r*), indicating how they were calculated

Our web collection on statistics for biologists contains articles on many of the points above.

Software and code

Policy information about availability of computer code

Data collection	The genetic instruments for circulating IL concentration were provide from the previously published journal (Bouras, E. et al, BMC Med 20,3 (2022)), and outcome GWAS summary statistics for kidney function traits were collected from the CKDGen database (URL: https://ckdgen.imbi.uni-freiburg.de/).
Data analysis	The summary level MR analysis was performed using the "TwoSampleMR" packages in R (version 0.4.26)

For manuscripts utilizing custom algorithms or software that are central to the research but not yet described in published literature, software must be made available to editors and reviewers. We strongly encourage code deposition in a community repository (e.g. GitHub). See the Nature Portfolio guidelines for submitting code & software for further information.

Data

Policy information about availability of data

All manuscripts must include a data availability statement. This statement should provide the following information, where applicable:

- Accession codes, unique identifiers, or web links for publicly available datasets
- A description of any restrictions on data availability
- For clinical datasets or third party data, please ensure that the statement adheres to our policy

All data used in this work are presented in the supplementary information that accompany the manuscript and are available in the original publications. The data used in this study is publicly available on the consortium website of the CKDGen (URL: https://ckdgen.imbi.uni-freiburg.de/)

Human research participants

Policy information about studies involving human research participants and Sex and Gender in Research.

Reporting on sex and gender	We performed MR analysis using genetic instruments and GWAS meta analysis data that do not provide sex-specific data. Therefore, we believe that the reporting on sex and gender is not applicable to our study.
Population characteristics	The summary statistics utilized in our study were based on UK biobank and CKDGen GWAS meta-analysis.<br>We reported the mean age and proportion of CKD in UK Biobank, and the median age and median of mean eGFR values in GKDGen meta-analysis.
Recruitment	As we utilized publicly available anonymous database of UK biobank and CKDGen, participant recruitment was not needed.
Ethics oversight	The institutional Review Board of the Seoul National University Hospital approved this study.

Note that full information on the approval of the study protocol must also be provided in the manuscript.

Field-specific reporting

Please select the one below that is the best fit for your research. If you are not sure, read the appropriate sections before making your selection.

☒ Life sciences ☐ Behavioural & social sciences ☐ Ecological, evolutionary & environmental sciences

For a reference copy of the document with all sections, see nature.com/documents/nr-reporting-summary-flat.pdf

Life sciences study design

All studies must disclose on these points even when the disclosure is negative.

Sample size	Four summary statistics for kidney function traits were utilized for MR analyses: 1) creatinine-based log-eGFR values of the CKDGen and the UKB data, 2) creatinine-based log-eGFR values from the phase 4 CKDGen study, 3) cystatin C-based log-eGFR values from the CKDGen and UKB, and 4) degree of annual eGFR decline including CKDGen and UKB.
Data exclusions	No data were excluded from the analyses.
Replication	Among above datasets, the 2) and 3) datasets were used as validation analyses for the main analysis with 1) dataset.
Randomization	Mendelian randomization analysis uses genetic instruments to control confounding effects.<br>A conventional randomization is not performed, however, as an assumptions for random genetic allocation, the method provides causal estimates.
Blinding	Blinding process was not relevant in this study.

Reporting for specific materials, systems and methods

We require information from authors about some types of materials, experimental systems and methods used in many studies. Here, indicate whether each material, system or method listed is relevant to your study. If you are not sure if a list item applies to your research, read the appropriate section before selecting a response.

Materials & experimental systems

n/a	Involved in the study	
☒	☐	Antibodies
☒	☐	Eukaryotic cell lines
☒	☐	Palaeontology and archaeology
☒	☐	Animals and other organisms
☒	☐	Clinical data
☒	☐	Dual use research of concern

Methods

n/a	Involved in the study	
☒	☐	ChIP-seq
☒	☐	Flow cytometry
☒	☐	MRI-based neuroimaging
